# Involuntary crying episodes with Susac’s syndrome—a rare presentation of a rare disease: a case report

**DOI:** 10.1186/s12883-022-02639-9

**Published:** 2022-04-25

**Authors:** O. Alshaqi, T. Moodie, A. Alchaki

**Affiliations:** 1 Damascus University, Damascus, Syria; 2 University of South Dakota, Vermillion, USA

**Keywords:** Susac’s syndrome, Hearing loss, Retinal artery occlusion, Involuntary crying, Pseudobulbar effect, Pseudobulbar laughing or crying, Pathologic weeping or crying or laughing, Emotional incontinence, Emotional dysregulation, Compulsive laughing or crying

## Abstract

**Background:**

In this case, we reported the pseudobulbar affect (PBA) in a patient with Susac’s syndrome—a rare condition that was caused by a rare syndrome. Previous case reports of Susac syndrome described psychiatric symptoms such as emotional disturbances or personality changes. Only a few case reports have reported psychiatric disorders in patients with Susac’s syndrome. There were no reported cases of Susac syndrome with PBA as an initial presentation.

**Case presentation:**

Our patient was 56 years old and presented with involuntary crying, left-sided headache, left-sided hearing loss, and tinnitus. Brain MRI showed numerous areas of restricted diffusion and enhancement involving the corpus callosum, bilateral hemispheres, and brainstem. Ophthalmological evaluation showed bilateral branch retinal artery occlusion. She was diagnosed with Susac’s syndrome and PBA. She was treated with cyclophosphamide and dextromethorphan hydrobromide/quinidine sulfate with excellent recovery. This is a 2-year clinical course.

**Discussion and conclusions:**

Recognition of the clinical presentation of Susac’s syndrome and PBA with early diagnosis and treatment are the keys to preventing further disability and impact on patients and their families.

## Background

Susac’s syndrome is an endothelial autoimmune disease. The immune system attacks the microvasculature (capillaries, venules, and arterioles) in the brain, retina, and inner ear (cochlea). It is characterized by a set of three main symptoms: encephalopathy, visual disturbance, and sensorineural hearing loss [1].

Accurate incidence and prevalence have not yet been determined. The medical literature has recently become more aware of Susac’s syndrome. The first case was reported in 1979 by JO Susac. Up to 2013, there were 304 reported cases worldwide. The syndrome is considered rare. However, the prevalence is believed to be higher because it is commonly misdiagnosed [2]. The target group is commonly considered to be young Caucasian nonpregnant women (20–40 years). Even so, it can affect pregnant women, children, elderly individuals, those of different races, and men [3].

There have been no worldwide definitive criteria for the diagnosis of Susac’s syndrome, at least until the moment of publication of this case report. However, a systematic review was published in 2016 to establish diagnostic criteria. The criteria were based on CNS, retinal and vestibulocochlear involvement [4].

Psychiatric disorders such as bipolar disorder have been reported in patients with Susac’s syndrome. PBA or emotional incontinence is a psychiatric disorder. It is described as uncontrollable outbursts of laughter or tearfulness. It is caused by bilateral corticobulbar tract degeneration. PBA has been reported in multiple neurological diseases, such as amyotrophic lateral sclerosis, Parkinson’s disease, dementia, and stroke. PBA has never been reported as an initial presentation of Susac syndrome.

## Case presentation

A 56-year-old woman presented to the emergency department (ED) with episodes of involuntary crying, dizziness, and decreased hearing over the previous 3 weeks. Prior to the emergency department visit, she had been treated by her primary care practitioner with prednisone and meclizine without improvement. Past medical history included seropositive rheumatoid arthritis (RA) and obesity. Her RA was well controlled with oral methotrexate. During the clinical encounter, she had uncontrollable outbursts of crying after each question that seemed exaggerated relative to her emotion. Each crying episode lasted for 2–5 min, and her mood returned to normal in between. Her neurological exam revealed nystagmus on the right lateral gaze.

**Fig. 1:**
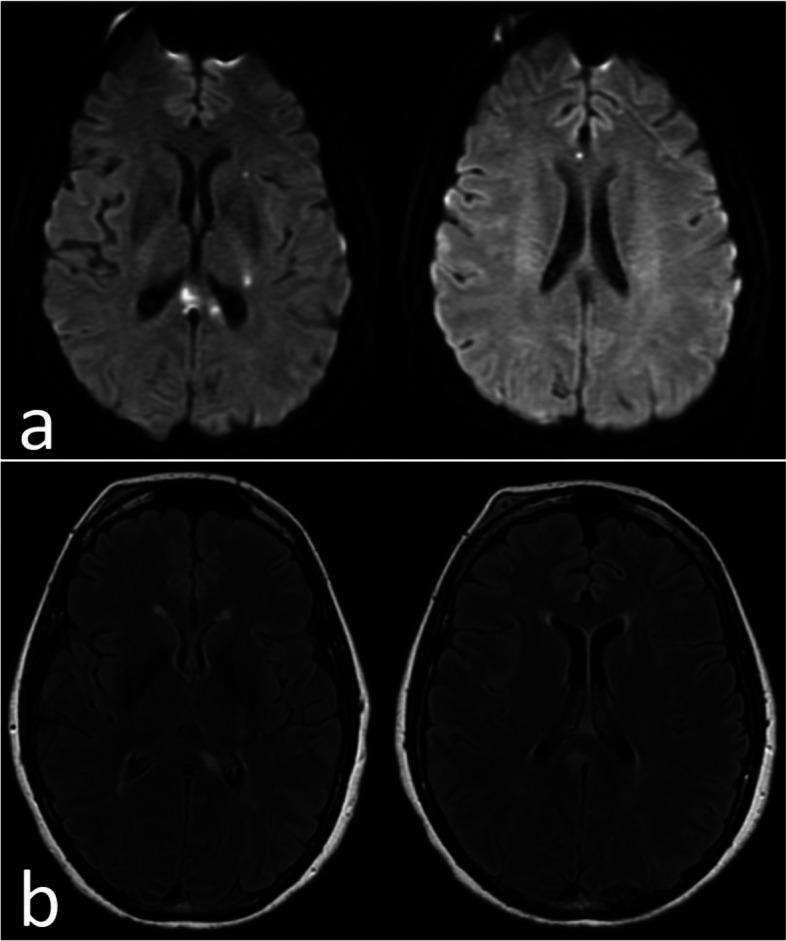
Initial brain MRI: (**a** DWI: showed areas of restricted diffusion over the central/left aspect of the splenium of the corpus callosum, left parietal white matter, left basal ganglia, and anterior right cingulate gyrus. **B** FLAIR: showed increased T2 signal in the corresponding areas)

Magnetic resonance imaging (MRI) of the brain showed areas of restricted diffusion over the central/left aspect of the splenium of the corpus callosum, left parietal white matter, left basal ganglia, and anterior right cingulate gyrus (Fig. 1). The initial etiology was determined to be embolic with an unknown source. Computed tomography angiography was negative. Echocardiogram was unremarkable. Loop recorder did not detect atrial fibrillation. Hypercoagulable workup was negative. Serum neuromyelitis optica and myelin oligodendrocyte glycoprotein were negative. The patient was discharged on dual antiplatelet therapy.

**Fig. 2:**
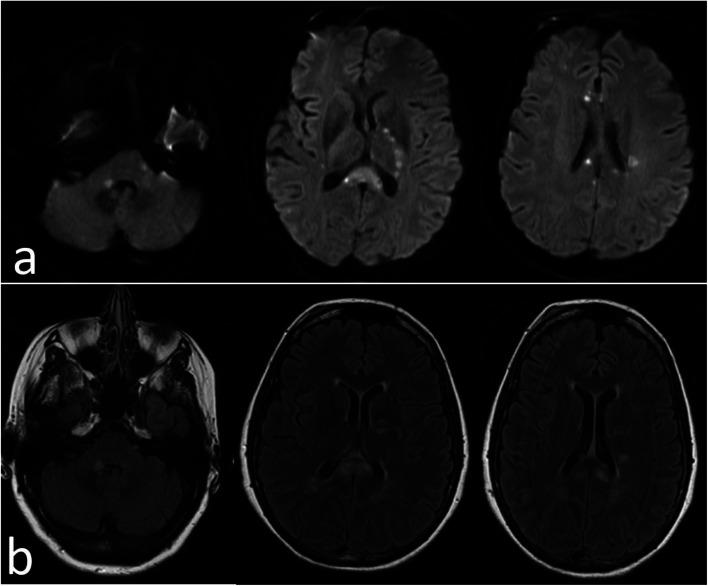
MRI brain 2 weeks later: (**a** DWI: new area of diffusion restriction in the Left corona radiata and expansion into the corpus callosum and right middle cerebral peduncle. **B** FLAIR: There was an area of increased T2 signal without diffusion restriction over the left side of the pontine)

She returned in 2 weeks with acute confusion, slurred speech, and headache. She continued to have decreased hearing and tinnitus in the left ear. On examination, she was disoriented and encephalopathic. Repeat brain MRI showed a new area of diffusion restriction in the left corona radiata and expansion into the corpus callosum and right middle cerebral peduncle. There was an area of increased T2 signal without diffusion restriction over the left side of the pontine (Fig. 2). Cerebrospinal fluid (CSF) evaluation showed a protein level of 277 mg/dl (normal range 12–60) and 2 white cells; otherwise, all results were normal (culture, cytology, meningitis encephalitis panel, ACE, myelin basic protein, oligoclonal bands, autoimmune encephalitis panel, N-methyl-D-aspartate receptor (NMDA), West Nile virus). The electroencephalogram (EEG) revealed no seizure activity. The cerebral angiogram was negative for vasculitis.

The patient received 5 days of intravenous immunoglobulin (IVIG) and methylprednisolone, which improved the overall clinical presentation. Approximately 1 week after completing steroids, the IVIG patient started developing new symptoms, including diplopia and internuclear ophthalmoplegia, with brain MRI showing a new area of diffusion restriction and enhancement.

The patient had no subjective visual loss, but an ophthalmological evaluation was requested based on suspicion of Susac’s syndrome given the corpus callosum lesions and hearing loss. The ophthalmological evaluation showed branch retinal artery occlusion. This finding led to the diagnosis of Susac’s syndrome.

After the diagnosis of Susac’s syndrome had been made, she was started on cyclophosphamide and slow prednisone taper. Additionally, she was found to be positive for hepatitis B, so tenofovir was started. She received cyclophosphamide IV monthly for 6 months. Then, six additional cyclophosphamide infusions were given, one every other month. Then, there were two further infusions once every 3 months. Cyclophosphamide was then discontinued. She remained on oral methotrexate for RA during the cyclophosphamide course.

**Fig. 3:**
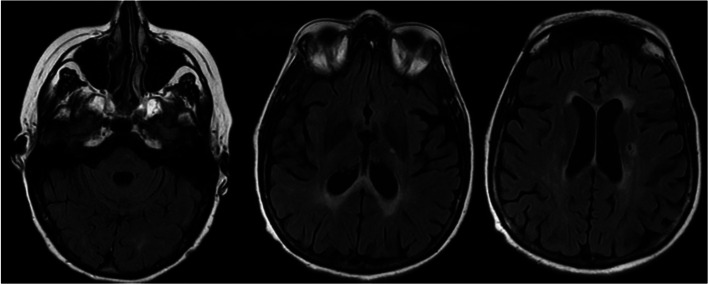
18 months later: (FLAIR: generalized brain atrophy with stable white matter lesions)

She had one relapse a month after starting cyclophosphamide, and she has been clinically stable since then. Follow-up brain MRI 18 months after her initial presentation showed generalized brain atrophy with stable white matter lesions. There were no diffusion-restricted or diffusion-enhancing lesions (Fig. 3). Her cognitive function and her mobility improved. Her pseudobulbar affect has been controlled with dextromethorphan HBr and quinidine sulfate. She has a hearing aid for her hearing loss.

## Discussion and conclusion

Susac’s syndrome is a rare, poorly characterized disorder. The diagnosis of Susac syndrome is based on a clinical presentation, a detailed medical history, and a variety of workups. Affected people may present with one or more of the triad of symptoms: encephalopathy, retinopathy, and inner ear symptoms [2]. Retinopathy can lead to dark spots, vision disturbance, or peripheral vision loss, or it may be asymptomatic because the occlusion is often located in the periphery of the retina. Poor circulation to the ear can lead to hearing loss, tinnitus, or vertigo [2]. This classic triad of symptoms were seen at disease onset in only 13% of cases [2]. A variety of additional neurological symptoms may develop, including confusion, generalized hypotonia, cognitive impairment, mood changes, and psychiatric symptoms [2]. In this case, our patient initially presented with hearing loss and pseudobulbar affect. Later, she developed encephalopathy, and retinopathy was only discovered during subsequent investigation.

The diagnostic workups include a brain MRI scan, retinal fluorescein angiography (FA), and audiometry. T2-weighted MRI can show characteristic changes in the brain, mainly the corpus callosum. Typically, the lesions are blurry (snowball lesions), which later convert into sharply defined (punched out) lesions. These lesions may be found in the periventricular region, cerebellum, brain stem, and deep gray matter nuclei. FA can confirm branch retinal artery occlusion and fluorescein leakage, often in the retinal periphery. Audiometry analysis can reveal a degree of unilateral or bilateral sensorial hearing loss. Other tests include cerebrospinal fluid analysis, which may show moderate protein elevation. Antiendothelial cell antibodies have been reported in 13% of patients [4].

The pathology of Susac syndrome is still unclear, but a recent study found that circulating CD8+ T cells were activated, clonally expanded, and differentiated into activated granzyme B- and perforin-expressing cytotoxic CD8+ T cells. Immunohistochemistry of brain tissue biopsy revealed the presence of cytotoxic CD8+ T lymphocytic infiltrates and thickening of the basal membrane, which led to microvascular wall proliferation. This led to occlusion, which ended with microinfarcts and the loss of neurons, axons, and myelin [5].

To date, no evidence-based treatment strategies exist. Early, aggressive treatment of Susac syndrome is recommended to prevent neurologic damage. The treatment is based on immunosuppressive medication. A high dose of glucocorticosteroids is effective in the acute phase. Long-term immunosuppressive treatments include monoclonal antibodies such as rituximab, intravenous immunoglobulin, cyclophosphamide, mycophenolate mofetil, and azathioprine [6]. Some studies have recommended administering a blood thinner, such as aspirin, and avoiding oral contraceptives and estrogen-replacement therapies to prevent blockage of blood vessels. Our patient had multiple relapses within a month and developed brain atrophy within a year. Early recognition and aggressive treatment result in a better prognosis and less disability.

The prognosis of Susac syndrome follows a different path for every patient and depends on the residual symptoms after each episode. It may range from full recovery to severe neuropsychiatric or physical disability. The disease usually lasts between 2 and 4 years. Some patients recover in a few months, even without treatment. Other patients continue to relapse after long-term treatment. There are a few reported cases in the literature of people who have died from the disease; however, death is not caused by the disease but caused by treatment complications.

Pseudobulbar affect is an unpredictable and uncontrollable emotional episode disconnected from or aggregated with the current mood state. This inappropriate reaction causes a significant emotional load on patients and caregivers if left untreated. The pathology of PBA is still unspecified. However, it is always correlated with brain damage or injury [7]. There is a marked degree of injury to the brain in patients with Susac’s syndrome [1]. The ability to express the appropriate emotion necessitates the presence of healthy complex neurocircuitry and neurochemistry [8]. From an anatomical standpoint, suitable emotions require connections across several areas of the brain, including the motor, prefrontal, and somatosensory cortices, limbic regions, the basis pontis, and the cerebellum (cortico-pontocerebellar circuit) [8]. Based on this understanding, one notion has been that any interruption in this network could result in PBA [8]. Our patient’s cingulate gyrus damage could explain her PBA.

On the other hand, serotonin, dopamine, glutamate, and sigma receptor type one (σ-1) play important roles in the regulation of emotions [9]. There was a study that showed lower serotonin receptor density in the midbrains of patients with PBA than in those without PBA [9]. Glutamate, an excitatory neurotransmitter, has also been implicated in the pathophysiology of PBA. The fact that the only medicine approved by the US Food and Drug Administration (FDA) for symptom treatment of PBA, dextromethorphan HBr and quinidine sulfate, is considered to operate on N-methyl-D-aspartate (NMDA) receptors emphasizes the role of glutamate in PBA [9].

## Data Availability

Not applicable.

## Abbreviations

ED: Emergency department
PBA: Pseudobulbar affect
RA: Rheumatoid arthritis
CSF: Cerebrospinal fluid
MRI: Magnetic resonance imaging
NMDA: N -methyl-D-aspartate
EEG: Electroencephalogram
FDA: US Food and Drug Administration
